# Accelerate Clinical Trials in Charcot-Marie-Tooth Disease (ACT-CMT): A Protocol to Address Clinical Trial Readiness in CMT1A

**DOI:** 10.3389/fneur.2022.930435

**Published:** 2022-06-27

**Authors:** Katy Eichinger, Janet E. Sowden, Joshua Burns, Michael P. McDermott, Jeffrey Krischer, John Thornton, Davide Pareyson, Steven S. Scherer, Michael E. Shy, Mary M. Reilly, David N. Herrmann

**Affiliations:** ^1^Department of Neurology, University of Rochester, Rochester, NY, United States; ^2^Faculty of Medicine and Health and Children's Hospital at Westmead, The University of Sydney School of Health Sciences, Sydney, NSW, Australia; ^3^Department of Biostatistics and Computational Biology, University of Rochester, Rochester, NY, United States; ^4^Health Informatics Institute, Morsani College of Medicine, University of South Florida, Tampa, FL, United States; ^5^Centre for Neuromuscular Diseases, Department of Neuromuscular Diseases, UCL Queen Square Institute of Neurology, London, United Kingdom; ^6^Department of Clinical Neurosciences, Fondazione IRCCS Istituto Neurologico Carlo Besta, Milan, Italy; ^7^Department of Neurology, Perelman School of Medicine at the University of Pennsylvania, Philadelphia, PA, United States; ^8^Department of Neurology, Carver College of Medicine, University of Iowa, Iowa City, IA, United States

**Keywords:** Charcot-Marie-Tooth disease (CMT), clinical trials, protocol, clinical outcome assessments, biomarkers

## Abstract

With therapeutic trials on the horizon for Charcot-Marie-Tooth type 1A (CMT1A), reliable, valid, and responsive clinical outcome assessments and biomarkers are essential. Accelerate Clinical Trials in CMT (ACT-CMT) is an international study designed to address important gaps in CMT1A clinical trial readiness including the lack of a validated, responsive functional outcome measure for adults, and a lack of validated biomarkers for multicenter application in clinical trials in CMT1A. The primary aims of ACT-CMT include validation of the Charcot-Marie-Tooth Functional Outcome Measure, magnetic resonance imaging of intramuscular fat accumulation as a lower limb motor biomarker, and *in-vivo* reflectance confocal microscopy of Meissner corpuscle sensory receptor density, a sensory biomarker. Initial studies have indicated that these measures are feasible, reliable and valid. A large prospective, multi-site study is necessary to fully validate and examine the responsiveness of these outcome measures in relation to existing outcomes for use in future clinical trials involving individuals with CMT1A. Two hundred 15 adults with CMT1A are being recruited to participate in this prospective, international, multi-center study. Serial assessments, up to 3 years, are performed and include the CMT-FOM, CMT Exam Score-Rasch, Overall Neuropathy Limitations Scale, CMT-Health Index, as well as nerve conduction studies, and magnetic resonance imaging and Meissner corpuscle biomarkers. Correlations using baseline data will be examined for validity. Longitudinal analyses will document the changes in function, intramuscular fat accumulation, Meissner corpuscle sensory receptor density. Lastly, we will use anchor-based and other statistical methods to determine the minimally clinically important change for these clinical outcome assessments and biomarkers in CMT1A. Reliable, and responsive clinical outcome assessments of function and disease progression biomarkers are urgently needed for application in early and late phase clinical trials in CMT1A. The ACT-CMT study protocol will address this need through the prospective, longitudinal, multicenter examination in unprecedented detail of novel and existing clinical outcome assessments and motor and sensory biomarkers, and enhance international clinical trial infrastructure, training and preparedness for future therapeutic trials in CMT and related neuropathies.

## Introduction

Charcot-Marie-Tooth disease (CMT) is a family of rare inherited peripheral neuropathies affecting ~1:2,500 individuals. CMT1A accounts for 50% of all people with CMT and is caused by an intrachromosomal duplication in chromosome 17 that results in the overexpression of peripheral myelin protein 22 kDa (PMP22) (1, 2). CMT1A is characterized by progressive weakness, imbalance, sensory loss, foot drop, and gait abnormalities resulting in reduced health-related quality of life (HRQoL) (3, 4). In the absence of pharmacologic interventions, treatment mainly consists of symptom management rehabilitation and surgical strategies. Research efforts, most recently, focus on disease modifying treatments including targeting correcting PMP22 overexpression using antisense oligonucleotides, small interfering RNA or small molecules as candidate therapies (5). Other therapeutic targets that have demonstrated preclinical or early clinical promise include inhibition of P2X7 receptor overexpression (6), neurotrophin 3 administration via gene therapy (7), and PP1R15A inhibition (to prolong unfolded protein response and reduce cell stress) (8). Most recently, a trial of a myostatin inhibitor did not demonstrate efficacy in regards to functional improvements (9).

Previously, ascorbic acid was thoroughly evaluated in individuals with CMT1A, with no benefit detected in multiple clinical trials (10–12). The primary outcome measure for the trials of ascorbic acid, the CMT Neuropathy Total Score (CMNTS), did not show significant change over a 2 year period in adults. These studies highlighted the need for additional natural history data as well as the development of reliable, responsive and clinically meaningful outcome assessments (COAs) in order for the CMT community to demonstrate clinical trial readiness (13). The Inherited Neuropathy Consortium (INC) has since done much work developing outcome measures and collecting natural history on the many different sub-types of CMT. As part of this work, the CMTNS was revised as a measure of disease severity to the CMTNS Version 2 (CMTNSv2) and then underwent Rasch analysis (CMTNSv2-R) (14, 15). Functional scales including the CMT Pediatric Scale (CMTPedS) and CMT Infant Scale (CMTInfS), have been developed and validated (16, 17). Using data from the INC, the CMTPedS detected disease progression in children with CMT1A over a 2 year period (18). Lastly, patient reported outcomes (PROs) to elucidate the individual's perspective of their disease have been developed and validated (19). Despite these achievements, there are still gaps in COAs for measuring the impact that CMT has on function. To address this limitation, we have developed and piloted the CMT-FOM (CMT-FOM), a measure for adults with CMT, modeled on the validated CMTPedS (20, 21). The CMT-FOM assesses physical function, including hand function, leg function/mobility and balance, areas that have been identified as having an impact on quality of life in individuals with CMT (22). This measure, once validated, will fulfill a critical need for future clinical trials in CMT as well as address a key element identified by the FDA for regulatory approval.

**Figure 1:**
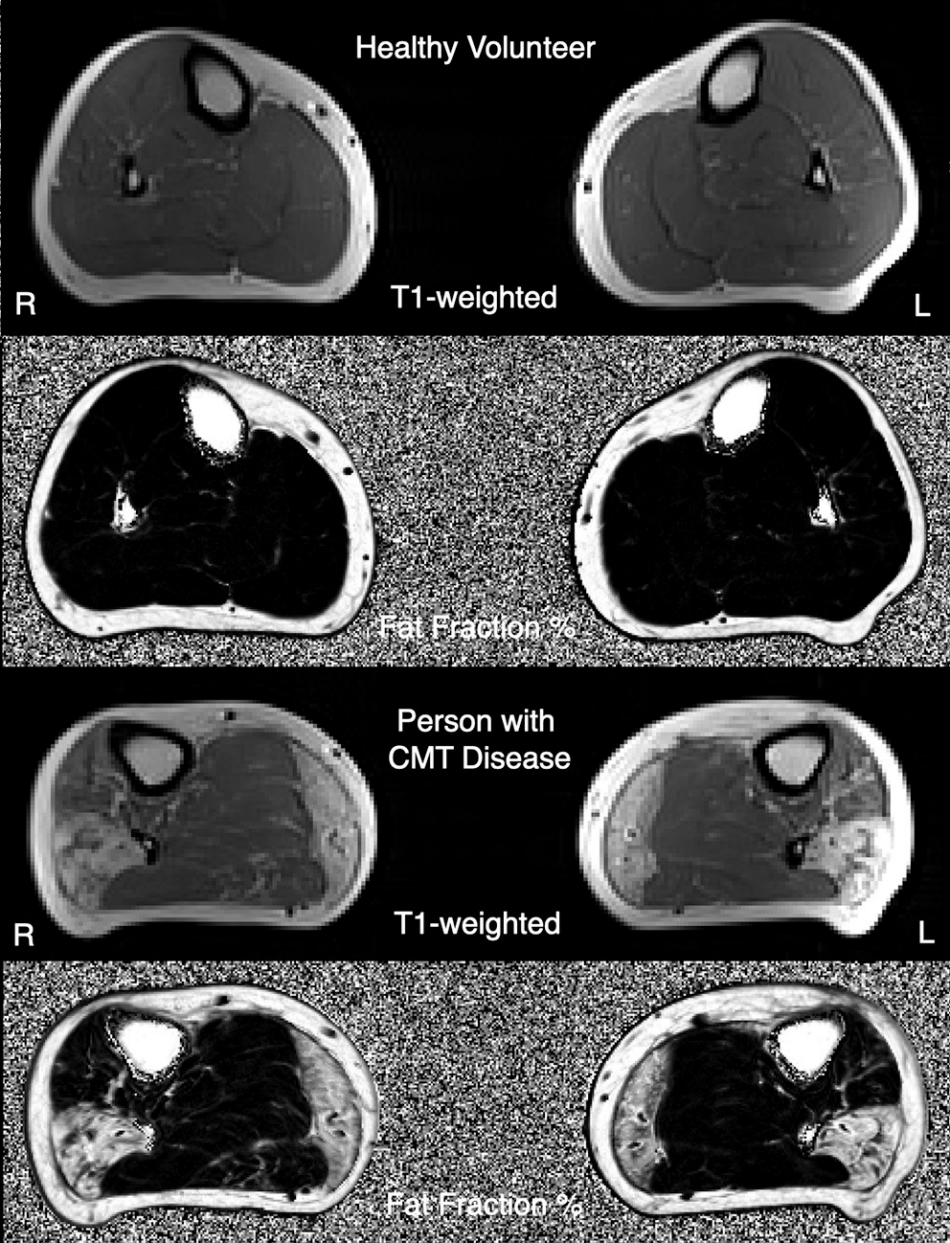
Example T1-weighted MRI images, and three-point Dixon MRI-obtained quantitative fat-fraction maps, from a healthy volunteer **(top images)**, and a person with CMT1A **(bottom images)**.

Beyond the need for COAs that are responsive to change, there is also a need for complementary biomarkers including target engagement measures and measures of treatment response that can be used in early phase clinical trials. Specifically, magnetic resonance imaging (MRI) to measure intramuscular fat accumulations (IMFA) in the calf (Figure 1) (23, 24) has, in pilot studies, detected disease progression in CMT1A over 12 months, the most responsive assessment to date in this disorder. Sensory dysfunction contributes significantly to functional impairment and reduced quality of life in CMT1A, yet objective measures of sensory dysfunction are lacking in CMT1A, with sensory nerve action potentials often unelicitable. *In vivo* reflectance confocal microscopy is non-invasive, and painless and has detected reductions of Meissner corpuscle density (RCM of MC density) in CMT1A that correlate with elevations of touch pressure sensory thresholds and clinical severity (25, 26). These motor and sensory biomarkers require longitudinal, multicenter validation prior to application in CMT1A clinical trials.

Therefore, the goal of the Accelerate Clinical Trials in CMT study (ACT-CMT) is to validate COAs and biomarkers for use in clinical trials involving adults with CMT1A. Specifically, we will evaluate the reliability, examine the construct and convergent validity, and document responsiveness to disease progression of the CMT-FOM. Similarly, using quantitative three-point Dixon MRI, we will examine the reliability, convergent validity and responsiveness to change of IMFA as measured by muscle fat fraction (FF) to validated it as a biomarker of calf-level muscle involvement, and last, we will assess the reliability, construct and convergent validity and ability to detect change of RCM of MC density to validate it as a sensory disease progression biomarker for use in multi-site CMT1A trials. We also aim to increase the efficiency of trial design by using data to refine eligibility criteria.

## Methods

### Study Description

The ACT-CMT study is a prospective, international, multi-center study of 215 individuals over a 3-year period. The University of Rochester, University of Iowa, and University of Pennsylvania are the enrolling sites in the United States and the European sites are University College London, UK and Fondazione IRCCS Istituto Neurologico Carlo Besta in Milan, Italy. The University of Rochester is the overall coordinating center for the study, providing training and quality assurance for all COAs, and is serving as the central training, quality assurance and reading core for blinded quantitation of MC densities from RCM image sets. The University College of London (UCL) MRC Neuromuscular Disease Centre is the central site for training, quality assurance and analysis of MRI of IMFA. The University of South Florida is the Data Management Center, and the University of Sydney will provide expertise in COA training and Rasch modeling for the CMT-FOM.

### Training and Quality Assurance

Principal investigators (PIs), study coordinators and clinical evaluators (CEs) attended an Investigators Meeting prior to the initiation of the study and participant enrollment. Study personnel received detailed procedure manuals for all assessments. Standardization and training in the administration and scoring of the CMTNSv2 was performed with the PIs and CEs. Following the meeting, the CEs participated in a hands-on training for administering the CMT-FOM. Following this training, evaluators assessed 10 individuals over 2 days to examine reliability (27). In addition to the training for the MRI assessments, the central MRI Reading Center (UCL) approved inter–scan reproducibility of control subjects at each site prior to beginning MRI assessments. MRIs are reviewed for image quality by the UCL MRI core with ongoing feedback to sites, and remedial steps as needed to rapidly address any data quality issue.

**Figure 2:**
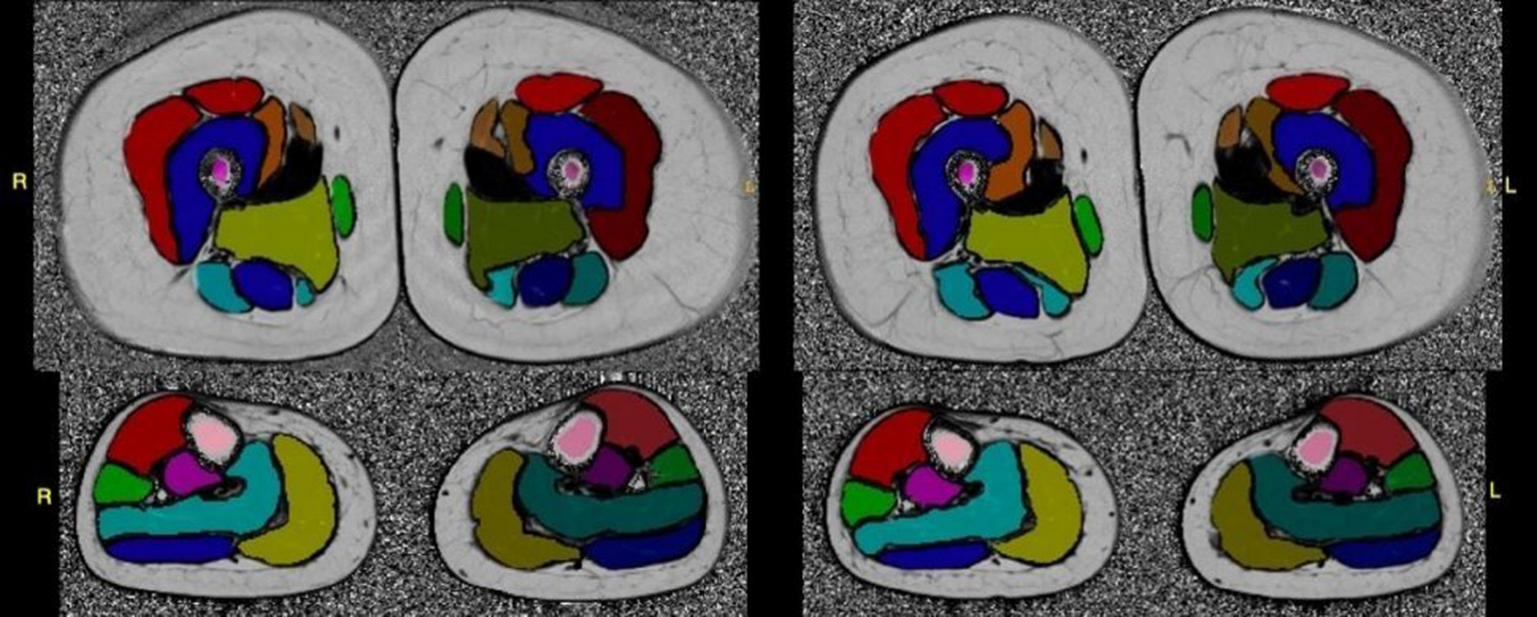
Test-retest fat-fraction map images from an example healthy volunteer for the calculation of reproducibility metrics. Images were acquired at two time points, 2 weeks apart. The fat fraction map was calculated from three-point Dixon acquisitions using the MRI study protocol, and shown with the placement of the whole muscle ROIs overlaid on the slice. All images were windowed to the same range for display (0–100%).

For RCM imaging, the University of Rochester RCM Core provided initial training for procurement of RCM image sets at the in-person meeting. Additional study personnel were trained by peers and remotely with real time feedback by RCM reading center staff using the microscope's TeamViewer application. Image sets are uploaded to the RCM Core and reviewed for image quality with ongoing feedback to sites (Figure 2).

### Recruitment of Participants

Subjects with CMT1A are primarily being recruited through the hereditary neuropathy and neuromuscular clinics led by the investigator at each site. Additionally, CMT1A subjects participating in the INC Rare Disease Clinical Research Network (RDCRN) studies at these sites are also given the opportunity to participate in the ACT-CMT study. An Institutional Review Board approved letter may also be sent to the participants of the INC RDCRN contact registry to inform them of the study and lastly, information about the study is posted on the websites of patient advocacy groups including CMT Association and the Hereditary Neuropathies Foundation. Control participants for this study are being recruited via postings in common areas at the sites that are accessible to a variety of individuals who travel through those areas. Control participants undergo MRI and RCM assessments only.

### Study Population

Individuals between the ages of 18 and 75 years with mild to moderate CMT1A will be recruited to participate in this study. This age range was selected as it is the age range that mirrors what is anticipated to be used in future adult CMT1A treatment trials.

In addition, all study participants are required to be ambulatory (assistive devices allowed) and have clinical and electrophysiological features of CMT1A, with a documented PMP22 gene duplication in the participant, or in an affected first degree relative, with documented nerve conduction slowing consistent with a diagnosis of CMT1A. The individuals are required to be able to participate in the informed consent process and document their consent. They must also be able to speak and read English (or Italian for the Italian site). Individuals are excluded from participation if they have other neuromuscular disorders, diabetes, exposure to peripheral neurotoxic agents, or other conditions known to predispose them to peripheral neuropathy. They are also excluded if foot or ankle surgery is planned or done within the 9 months preceding the screening, or have a medical condition that in the opinion of the investigator precludes participation in the CMT-FOM data collection. For the MRI study procedures, individuals are additionally excluded from participation if there is a contraindication for non-contrast MRI.

Healthy controls of the same age range participating in the MRI and/or RCM assessments are required to be able to provide written informed consent. They must also be able to speak and read English (or Italian for Italian site). Healthy control participants are required to meet the same exclusion criteria outlined above for participants with CMT1A. Additionally, individuals are excluded from participation if they have a family history of a known hereditary neuropathy, unless previously been shown to be negative for PMP22 gene duplication/deletion.

## Measures

To address the objectives of this study, participants complete assessments at baseline, and every 6 months for up to 36 months. Depending on the time of enrollment, participants will complete either a 30- or a 36-month end of study visit, but not both. Participants will be asked to complete the CMT-FOM, MRI and RCM assessments (Table 1). In addition, measures of disease severity and burden, sensory assessments, the fibular distal compound muscle action potential (CMAP) recording from the tibialis anterior muscle, and blood specimens will be collected. Participants will also complete a global impression of change questionnaire.

**Table 1:** Schedule of activities.

**Study visit**		**Screen**	**1**	**2**	**3**	**4**	**5**	**6**
Timeframe (months)		0	0	6	12	24	30	36
Activities for all participants	Informed Consent	x						
	Demographics	x						
	Medical history	x		x	x	x	x	x
	Concomitant medications	x		x	x	x	x	x
	CMTES	x	x	x	x	x	x	x
	CMTNSv2-R (CMTES + tadial SNAP and ulnar CMAP)*		x		x	x		x
	Peroneal [tibialis anterior (TA)] CMAP amplitude		x		x	x		x
	CMT-FOM (up to 10 subjects will also perform CMT-FOM reliability testing)		x	x	x	x	x	x
	PROs: CMTHI, ONLS, PGIC		x	x	x	x	x	x
	Adverse Events	x	x	x	x	x	x	x
	Blood draw for Biomarkers		x	x	x	x	x	x
Additional AIM 2 items	MRI thigh and calf muscle IMFA		x		x	x		
Additional AIM 3 items	RCM MC density digit V and thenar eminence		x	x	x	x	x	x
	Monofilament touch sensation threshold testing		x	x	x	x	x	x

*SNAP, sensory nerve action potential; CMAP, compound muscle action potential*.

^*^Nerve conduction studies will be conducted at 30 month visit when end of study for that subject.

*Subjects will have either visit 5 (30 months) or visit 6 (36 months) depending on when they enroll. They will not have both. Subjects will be informed at time of enrollment whether they will have a 30 or 36 month visit*.

### Charcot-Marie-Tooth Functional Outcome Measure

The CMT-FOM is a 13-item performance-based physical functioning measure. The items of the CMT-FOM assess upper and lower limb physical functioning, gait, mobility and balance. These items include the nine-hole peg test, functional dexterity test, grip dynamometry, 6-minute walk test (6MWT), 10-meter (10 m) walk/run test, time to climb 4 stairs, 30 second chair stand test, Timed Up and Go test (TUG), dynamometry for ankle dorsiflexion and plantarflexion, and balance items (standing feet apart on a line, eyes open, standing feet apart on a line, eyes closed, standing on one leg, eyes closed) (20, 21). Similar to the CMTPedS (16) and CMTInfS (17), items are scored by converting the raw data to a z-score based on normative data. The z-scores are then categorized as normal = 0, very mild = 1, mild = 2, moderate = 3 or severe = 4, based on the amount of deviation from normal, to generate a total score of 0–52 (21). The CMT-FOM takes ~35 mins to complete. Our initial study of the CMT-FOM demonstrated good test-retest reliability and an association with the CMTES, consisting of the clinical items of the CMTNSv2, providing evidence to support validity (21). As part of this validation study, inter-rater reliability was established following the initial evaluator training at the pre-study investigator meeting (27).

### MRI Assessment for Intramuscular Fat Accumulation

Initial pilot studies examined the utility of MRI to assess changes in muscle and identified fat fraction as a potential biomarker for studies in CMT1A. FF was found to correlate with the CMTES and demonstrated sensitivity to disease progression over a 12-month period (23). To validate this measure for multi-site use in future clinical trials, MRI muscle imaging of the lower extremities will be done using three-point Dixon MRI to assess the %FF of the bilateral thigh and calf muscles. Participants will be imaged feet-first supine with surface matrix coils for lower limb signal reception. Prior to participant enrolment, MRI studies of non-affected individuals will be performed and sent to a central site (UCL) for quality control and analysis (24). MRI scans for participants with CMT1A will also be sent to a central site for analysis.

### *In vivo* Reflectance Confocal Microscopy of Meissner's Corpuscles

**Figure 3:**
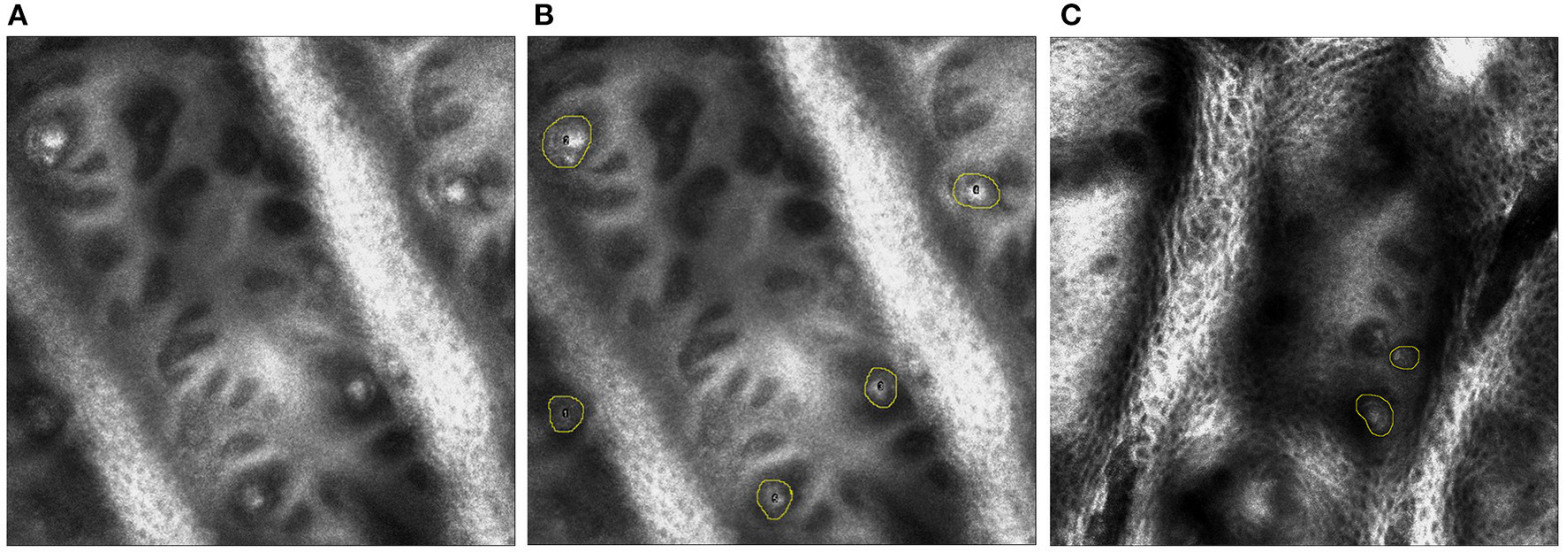
**(A)** On the left, single plane image from *in vivo* reflectance confocal microscopy of digit V. **(B)** In the middle, the MCs are identified by yellow circles in a healthy control. **(C)** On the right, MCs are identified by yellow circles in an individual with CMT 1A.

*In vivo* RCM of MC has been identified as a novel approach to measure sensory neuropathy (25, 26, 28). MC density of the fingertip of digit V was found to be lower than that of healthy controls and was associated with overall CMT disease severity. In this study, a portable *in vivo* confocal reflectance microscope (Vivascope 3000, Caliber Imaging and Diagnostics Inc.) will be used to assess MC density. *In vivo* RCM of MCs will be performed on the palmar surface of the distal phalanx of digit V and the thenar eminence of the hand. The non-dominant side will be used, unless there is a history of focal trauma, or entrapment neuropathy, that will confound or preclude imaging. In that instance, RCM and touch pressure sensation threshold testing will be done on the dominant side. The *in-vivo* RCM procedure involves applying a few drops of bio-compatible index matching fluid to the skin site, touching the window of the lens to the surface of the skin and obtaining a standardized set of images using the system's operating software (Figure 3).

### Additional Assessments

The CMTNS is a composite measure of disease severity that was developed for use in clinical trials (29). It includes items assessing sensory and motor symptoms, sensation (vibration and pinprick), distal limb strength and limited nerve conduction studies. Following the studies of ascorbic acid in CMT1A, (11, 12) it was modified to improve sensitivity and minimize floor and ceiling effects (CMTNSv2) (14). Most recently, Rasch analysis was applied resulting in the CMTNSv2-R (15). The CMT Exam score (CMTES-R) removes the ulnar motor (CMAP) and radial sensory action potentials (SNAP) amplitudes from the CMTNSv2-R and has been found to be more responsive than the CMTES (30).

Monofilament touch sensation threshold testing will be performed in all participants at the skin sites of *in vivo* RCM of MCs at the hand (digit V and the thenar eminence). Testing will be performed using a 2 Alternative Forced-Choice Stepping Algorithm (31) with a Logarithmic Filament Set. This filament set consists of a series of 9 monofilaments producing magnitudes of force of −3 to 5 in grams in natural log increments at 5/6 of their extended lengths. The nine levels of stimulus forces are available to define 19 threshold levels (32).

The Overall Neuropathy Limitation Scale (ONLS) is a clinician-administered assessment where participants are asked a series of questions about their symptoms and their ability to perform certain tasks and movements. This activity-level measure is designed to distinguish between limitations of upper and lower limbs in individuals with peripheral neuropathies (33).

The CMT Health Index (CMT-HI) is a patient-reported, disease-specific measure of disease burden that was developed for use in clinical trials involving adults with CMT (34). It has been shown to be reliable and valid and has been translated into Italian for multi-site clinical trial use (35).

The Patient Global Impression of Change (PGIC) questionnaire inquires about total health and health related to the symptomatic themes assessed in the CMT-FOM. Participants will be given the option to state that, compared to baseline, their health is: “1—a lot worse”; “2—a little worse”; “3—the same”; “4—a little better”; or “5—a lot better.” The PGIC will be used as an anchor to determine minimal clinically important change for the CMT-FOM.

## Sample Size

We are recruiting 215 individuals with CMT1A to participate in this study. This baseline sample will provide >90% power to detect correlations between the CMT-FOM and existing COAs and PROs as small as 0.22. We anticipate that 180 participants will complete the assessments at the 30-month visit (accounting for up to a 15% dropout). This sample size will provide >80% power to detect significance of a mean change of 0.21 standard deviation units (effect size) using a paired t-test. This effect size was selected as one that is larger than those of existing adult scales such as the CMTNS (0.13) and the CMTES-R (0.20) (36, 37).

A sample of 60 individuals with CMT1A subjects at baseline will provide > 80% power to detect correlations between muscle FF and clinical outcome variables as small as 0.35, using a two-tailed test and a 5% significance level. Anticipating a dropout of 15%, a sample size of 51 CMT1A subjects at 24 months will provide 90% power to detect significance of a mean change of 0.46 standard deviation (SD) units (absolute increase in FF of 0.69%) (23) using a paired t-test. Sample sizes of 51 CMT1A subjects and 21 controls will provide 80% power to detect a group difference in mean change of 0.65%, assuming standard deviations of 1.5% in the CMT1A group and 0.4% in controls (23), using a two-sample t-test. Lastly, for validating RCM of MC density as a sensory biomarker a sample size of 135 CMT1A subjects at baseline will provide >90% power to detect correlations between MC density and clinical outcome variables as small as 0.28. Again, it is anticipated that 115 participants will complete assessments at 30 months which will provide 90% power to detect significance of a mean change of 0.31 standard deviation units using a paired t-test (36).

## Statistical Considerations

### Validation of the CMT-FOM

Preliminary studies of the CMT-FOM have documented feasibility, content validity, and concurrent validity (21). This study will further validate the CMT-FOM by assessing and documenting inter-rater reliability, performing Rasch analysis, and examining convergent validity and responsiveness. We will also determine the minimal clinically important change. We examined the inter-rater reliability of the CMT-FOM following the investigator meeting and the results have been reported (27). Using baseline data, we will document the internal consistency of the CMT-FOM with Cronbach's alpha.

Given that the CMT-FOM was based on the extensive validation of the CMTPedS (16), we hypothesize that it will be unidimensional and measure the construct of functional ability. We will examine the dimensionality of the CMT-FOM using principal component analysis and confirmatory factor analysis. Misfitting items will be examined and potentially modified, prioritizing integrity of outcome and concept validity. Rasch analysis will be performed on the CMT-FOM, including assessment of the response format, fit of the items, item bias, unidimensionality, and spread of items across the construct being measured (37).

Using data from the baseline visit, correlation and regression analyses will be performed to examine convergent validity. Specifically, we will examine the associations between the CMT-FOM and measures of disease severity (CMTNSv2-R, CMTES-R), patient reported outcomes, such as the CMT-HI, as well as electrophysiological outcomes. We anticipate finding correlations between the CMT-FOM and existing CMT1A measures, but not so strong as to make the CMT-FOM redundant.

To examine sensitivity to change, longitudinal analyses will evaluate the CMT-FOM in all affected subjects evaluated at 0 (baseline), 6, 12, 24, 30 and 36 months. The effect size and standardized response mean (SRM) are the most highly recommended measures of responsiveness (38). These two measures will be computed for CMT-FOM change from baseline, as well as changes in the CMTNSv2-R, CMTES-R, PROs (CMT-HI, PGIC), and the biomarkers at each time point (38, 39). The effect sizes and SRM will be compared between measures using bootstrap resampling (40). Mixed model repeated measures (MMRM) analyses will model mean changes over time in these outcomes (41).

The minimal clinically important change of the CMT-FOM will be estimated by anchor-based and distribution-based methods (42, 43). Anchor-based methods will use the PGIC questionnaire. Mean changes in the CMT-FOM will be calculated at 12 and 24 months for subjects in each of the five categories. Receiver operating characteristic (ROC) curve analyses will be performed separately for the 12- and 24-month outcomes to identify the change in CMT-FOM that best discriminates subjects who stated that their health got “a little” or “a lot” worse compared to those who did not. The 12, 24, 30 and 36-mo. changes in the CMT-FOM that correspond to effect sizes ranging from 0.30 to 0.50 standard deviation units will be described and compared to the minimal clinically important change.

For IMFA, muscle FF values for each subject will be combined into a summary measure for all muscles (left and right limb) at thigh and at calf level, and for relevant functional groups (quadriceps, hamstrings, anterior tibial compartment, and triceps surae) for left and right limbs separately (23). Using baseline data, cross sectional differences in muscle FF between the CMT1A group and matched controls will be assessed using analysis of covariance with group, age, and gender included in the model; large group differences are expected. Associations between FF and the COAs will be evaluated using correlation and regression analyses in CMT1A subjects.

Longitudinal analyses will be performed to evaluate responsiveness to change over time of MRI quantified muscle FF in the 60 adults with CMT1A. MRI scans will be evaluated at baseline, 12 and 24 months. Effect sizes and standardized response means will be computed for muscle FF. MMRM analyses will be performed to model mean changes over time in these outcomes (41). We will explore possible predictors of change over time using these models by adding interaction terms between covariates (e.g., age, sex, baseline severity) and month to the models. Mean changes over time, with corresponding 95% confidence intervals and *p*-values, will be obtained from these models. The results may suggest targeted eligibility criteria in future studies that will identify subjects more likely to change over time and, hence, increase power. Change in FF will be correlated with changes in the above clinical and electrophysiologic outcomes in CMT1A subjects to provide longitudinal validation of the MRI biomarker. This will assess whether early changes in FF are associated with longer term functional outcomes.

Reliability of MC density at digit V and the thenar eminence in participants with CMT1A will be assessed using a sample of 20 RCM image sets and two reviewers. Intra-rater reliability will be quantified using intraclass correlation coefficients (ICCs), estimated using a one-way random effects analysis of variance model with participant treated as a random effect. Interrater reliability will be evaluated using ICCs estimated using a two-way random effects analysis of variance model with participant and rater treated as random effects. The ICCs should exceed 0.80; any lower values will motivate us to reevaluate the scoring process and/or perform further training of the raters.

Baseline associations between MC densities and the touch sensation thresholds and FF of the calf muscle will be examined using correlation and regression analyses. Also at baseline, differences between CMT1A subjects and controls with respect to mean MC density will be evaluated using analysis of covariance models that include group as well as age, gender, hand dimension (if appropriate), and height. For longitudinal data, MMRM analyses will be performed to model mean changes over time in the MC density outcomes (41). We will explore possible predictors of change over time using these models by adding interaction terms between covariates (e.g., age, sex, baseline severity) and month to the models. Mean changes over time, with corresponding 95% confidence intervals and *p* values, will be obtained from these models. Changes in MC density will be correlated with changes in the COAs, touch sensation thresholds, ONLS and PROs in CMT1A subjects to provide longitudinal validation of this sensory biomarker.

## Discussion

While CMT1A is the most common type of CMT, accounting for over 50 percent of cases, it is still a rare condition. CMT1A is slowly progressive; as such, it has proven challenging to measure disease progression with existing CMT outcome measures in the context of clinical trials. Multi-site studies are necessary to recruit sample sizes large enough to validate outcome measures and inform future clinical trial needs. The ACT-CMT study, has been designed to address these needs and prepare for future clinical trials in CMT1A. The eligibility criteria were selected to reflect the potential criteria for trials involving adults with CMT1A; however, the data from this study will likely be able to be leveraged to optimize eligibility criteria.

The CMT-FOM is hypothesized to be a unidimensional measure that with the CMTInfS and CMTPedS will provide whole of life CMT functional outcome measures. Initial studies have documented the reliability and supported the validity of this COA; however, this study will provide the sample size necessary for full validation, Rasch analysis, and examination of the ability of the CMT-FOM to detect disease progression over a time period that is feasible for the conduct of multi-center trials. The ACT-CMT protocol will also promote rigorous validation of existing COAs that have been used in prior trials in CMT (including the CMTES and the ONLS) for comparison with the CMT-FOM. Moreover, we have recently developed and reported cross-sectional validation of a disease specific patient reported outcome measure (CMT-HI) to capture patient reported disease burden in CMT (34). The ACT-CMT protocol will permit careful longitudinal validation of the responsiveness to change of the CMT-HI. Insight into clinically meaningful change in outcome measures for hereditary neuropathies is lacking. The current protocol will rigorously assess the clinically meaningful change of each COA.

Validated biomarkers of the motor and sensory components of CMT1A for multicenter trial application has been identified as a gap in clinical trial readiness. Given the slow progression of CMT1A, biomarkers that have capacity to demonstrate slowing of disease progression or improvement, prior to changes in physical functioning, neurologic impairment or symptoms, will enable go/no go decisions in early phase clinical trials in CMT1A. Indeed, data to date for lower extremity MRI of muscle FF, has suggested that this motor biomarker is highly responsive to change (standardized response mean >0.8) in CMT1A. The ACT-CMT protocol will establish the sensitivity to change of MRI of intramuscular FF and RCM of MC density. ACT-CMT will also determine whether these biomarkers can be implemented at multiple centers, and in the case of MRI, using MRI scanners from different manufacturers. This protocol will provide the data to determine whether changes in calf intramuscular FF and RCM of MC density predict changes on clinician administered and patient reported outcome measures in CMT1A. These data will support the implementation of these biomarkers in future trials in CMT1A.

Successful conduct of multicenter, transcontinental clinical trials in rare disorders such as CMT1A require the establishment of robust clinical trial site capacity, site training and certification in the administration of COAs and imaging, and data transfer procedures that meet data privacy standards. Moreover, efficient and reliable central image analysis cores are required for modalities such as MRI of IMFA and quantitation of RCM of MC densities. The ACT-CMT study will establish the multicenter capacity, expertise and quality assurance to allow for seamless transition from the clinical trial readiness study to implementation of clinical trials in CMT1A. The data that will emerge from this study will inform participant selection criteria, outcome measure and biomarker selection, sample size and trial duration considerations. These data will be made available to the scientific community and will inform discussions with regulatory agencies during early stages of drug development. Additionally, the ACT-CMT study will establish a large cohort of adults with well characterized CMT1A, which will accelerate recruitment for future therapeutic trials in CMT1A, and elucidate the natural history of CMT1A in unprecedented detail using a range of clinician administered and patient reported outcome assessments and biomarkers, supporting clinical trial design.

The ACT-CMT study has some potential limitations. This study is being conducted at sites in the United States, UK and Italy. The clinician administered and patient reported outcome measures are currently established for English and Italian speaking study personnel and participants. If validated, these COAs will need to be extended to other languages for utility in global clinical trials. The ACT-CMT protocol focuses on individuals 18–75 years of age, as the majority of individuals with symptomatic CMT1A are adults, and critical gaps exist in outcome measures for adults with CMT1A. However, as therapeutic effects will potentially be greatest with intervention prior to development of significant disability, early treatment in late childhood or adolescence will be critical for CMT1A. The CMTPedS, on which the CMT-FOM is modeled, has already been longitudinally validated for children and adolescents with CMT1A, and studies are proceeding in parallel with ACT-CMT through the INC to evaluate biomarkers. Additionally, the use of MRI of calf and foot muscle to evaluate FF in children with CMT1A is being examined at two of the ACT-CMT sites (London and Iowa). Therefore, the findings of ACT-CMT will need to be considered in conjunction with data emerging from these studies in children when planning future clinical trial programs in CMT1A.

We anticipate that the ACT-CMT study will fill critical gaps in the toolkit of outcome measures for early and late stage clinical trials in CMT1A and accelerate the pathway toward design of successful clinical trials. CMT1A has overlapping clinical features with other major forms of CMT including CMT1B, CMTX1 and other forms. The outcome assessments and motor and sensory biomarkers incorporated in the ACT-CMT study will likely meet the needs of clinical trials for other forms of CMT. This study, therefore, creates a framework that can be used to validate these and other measures in preparation for clinical trials in other subtypes of CMT.

## Data Availability Statement

The original contributions presented in the study are included in the article/supplementary material, further inquiries can be directed to the corresponding author/s.

## ACT-CMT Study Group

Mariola Skorupinska, Menelaos Pipis, Christopher Record, Luke O'Donnell, Magdalena Dudziec, Matilde Laura, Jasper Morrow, Amy McDowell, Carolynne Doherty, Chiara Pisciotta, Claudia Ciano, Domenico Aquino, Paola Saveri, Giulia Schirinzi, Daniela Calabrese, Timothy Estilow, Dragan Vujovic, Pooja Patel, Nidia Villalpando, Paige Howard, Riccardo Zuccarino, Valeria Prado, Peter Creigh, Steffen Behrens-Spraggins, Elizabeth Wood, Julie Charles, Kimberly Hart, Lindsay Baker, Paula Bray, Melissa Mandarakas.

## Author Contributions

DH involved in funding. KE involved in writing the manuscript. JS, JT, JB, DP, SS, MS, DH, KE, and MR are supervising the execution of the study. JS, JT, JB, DP, SS, MS, DH, KE, MM, JK, and MR are helping to design the study. MM and JK are performing data management and analysis. All authors reading and approving the final manuscript.

## Funding

DH was funded by NIH grant # NIH 1 U01 NS109403-01.

## Conflict of Interest

KE reports grant support through the Charcot-Marie-Tooth Association and has served on advisory boards for Biogen, Roche, Dyne, and has received consulting fees from Fulcrum Therapeutics, Dyne, Acceleron Pharma, Avidity and Roche. JB reports work in the Burns Group is supported by the Australian Government Department of Health (NHMRC-MRFF #1152226), US National Institutes of Health (NINDS #1U01NS109403-01, NINDS #U54NS065712), US Muscular Dystrophy Association (Idea Grant #876246), Charcot-Marie Tooth Association of Australia, Charcot Marie Tooth Association (USA), Cerebral Palsy Alliance, Diabetes Australia, Humpty Dumpty Foundation, Kids Neuroscience Centre, The Children's Hospital at Westmead, and The University of Sydney. Consultancies over last 18 months: Acceleron Pharma, Pharnext, Passage Bio, Inc. He is a registered podiatrist working at the Children's Hospital at Westmead, Sydney, Australia. JT reports consultancies with Invicro, LLC and F. Hoffmann-La Roche Ltd. DP reports grant support from Telethon-UILDM, AFM-Telethon, National Institutes of Health, and the Charcot-Marie-Tooth Association, serves on clinical advisory boards for Inflectis, Alnylam, and Akcea, Arvinas, and Augustine, received travel grants from Kedrion Spa and Pfizer, and Istituto Neurologico Carlo Besta receives donations for research from Pfizer, LAM Therapeutics, Acceleron Pharma Inc. SS actively serves as a consultant to Disarm Therapeutics, Mitochondria in Motion, and Pfizer and has research support from the National Institutes of Health, Muscular Dystrophy Association, and the Charcot-Marie-Tooth Association. He reports grant support from U54 NS0657, the Muscular Dystrophy Association, and the Charcot-Marie-Tooth Association. He also serves as a consultant for Inflectis BioSci, Alnylam Pharma, Mitochondria in Motion, Passage Pharma, Applied Therapeutics, and DTx Pharm. MR reports grant support from U54 NS0657, Muscular Dystrophy Association, Charcot-Marie-Tooth Association, the Medical Research Council (MRC) and the Wellcome trust and consults for Inflectis, Alnylam, Akcea and served on a steering committee for Eidos. He reports grant support through U54 NS065712, NINDS. 5U01NS109403-04, 1R01DK115687-04, the CMT Association, Muscular Dystrophy Association Friedreich's Ataxia Research Alliance, Acceleron Pharma. He also reports consulting fees from Regenacy Pharmaceuticals, Acceleron Pharma, Alnylam, Neurogene, Applied Therapeutics, Sarepta, Passage Bio, Pfizer Guidepoint Global, GLG, Slingshot Insights, ClearView Health Partners, MedPace, DDB Health NY, Cydan, Trinity Partners, Schlesinger. The remaining authors declare that the research was conducted in the absence of any commercial or financial relationships that could be construed as a potential conflict of interest.

## Publisher's Note

All claims expressed in this article are solely those of the authors and do not necessarily represent those of their affiliated organizations, or those of the publisher, the editors and the reviewers. Any product that may be evaluated in this article, or claim that may be made by its manufacturer, is not guaranteed or endorsed by the publisher.
